# Improved outcomes with robotic-assisted laparoscopic paraesophageal hernia repairs compared with laparoscopic and transthoracic approaches: A single high-volume institution experience

**DOI:** 10.1016/j.xjon.2025.06.016

**Published:** 2025-06-25

**Authors:** Katelyn R. Ward, Jenny Bui, Irina Bondarenko, Andrew Chang, Kiran Lagisetty, Jules Lin, Chigozirim N. Ekeke, David D. Odell, Rishindra M. Reddy

**Affiliations:** a Department of Surgery, Corewell Health East William Beaumont University Hospital, Royal Oak, Mich; b Section of Thoracic Surgery, Department of Surgery, University of Michigan, Ann Arbor, Mich; c Department of Surgery, Henry Ford Hospital, Detroit, Mich

**Keywords:** paraesophageal hernia, hiatal hernia, robotic-assisted laparoscopic, laparoscopic, transthoracic, radiographic recurrence, symptomatic recurrence, hernia recurrence

## Abstract

**Objectives:**

Laparoscopic (lap) paraesophageal hernia repair has excellent short-term outcomes but higher long-term recurrence rates compared with the transthoracic repair. We hypothesized that the robotic-assisted lap (robot) approach would have similarly good short-term outcomes as lap, but also lower recurrence rates.

**Methods:**

A retrospective study of prospectively collected data was performed for paraesophageal hernia repairs at a single high-volume quaternary hospital from July 2018 to September 2022. Outcomes analyzed included 2-year postoperative radiographic recurrence (Rad), Society of Thoracic Surgeons-defined radiographic recurrence (STS-rad), symptomatic recurrence (Sx), and perioperative outcomes. Lap, robot, and transthoracic groups were compared using univariate, multivariate, and propensity score analysis.

**Results:**

Among 207 cases (52 lap, 90 robot, and 65 transthoracic), robot was lower than lap (odds ratio [OR], 0.13-0.17; *P* < .01) and similar to transthoracic (OR, 0.79-1.02; *P* > .05) in univariate and multivariate analyses. STS-rad was similar between approaches across analyses, apart from robot being higher than transthoracic on propensity score analysis (OR, 1.83; *P* < .01). Robotic Sx recurrence was lower in robot compared with lap across analyses (OR, 0.40-0.50; *P* < .001). Median length of stay was 2 days for robot and lap, significantly shorter than transthoracic (median, 5 days; *P* < .01). Fewer postoperative complications occurred in robot compared with transthoracic (OR, 0.19-0.21; *P* < .01). Reoperation and endoscopic intervention were lower in robot compared with lap (OR, 0.09-0.12; *P* < .01 and OR, 0.32-0.40; *P* < .05).

**Conclusions:**

Robotic paraesophageal hernia repairs had generally lower 2-year recurrence and reoperation than lap and shorter hospital stays and fewer immediate complications than transthoracic.

**Figure:**
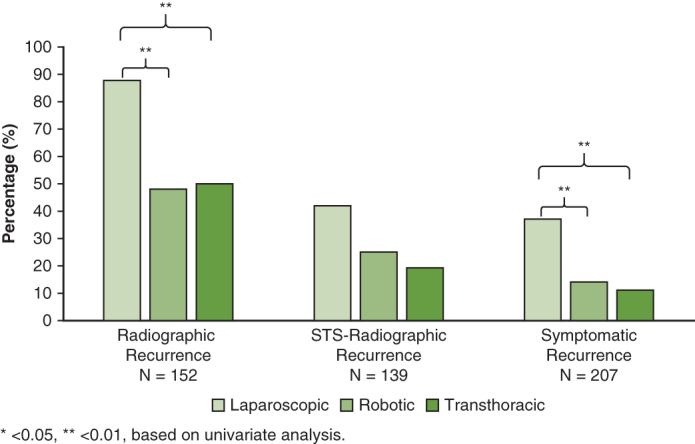
∗*P* < .05 and ∗∗*P* < .01, based on univariate analysis.

**Central Message**

Robotic laparoscopic paraesophageal hernia repair is associated with lower short-term morbidity compared with an open transthoracic approach, and reduced long-term recurrence compared with laparoscopic.

**Perspective**

The robot-assisted laparoscopic approach to paraesophageal hiatal hernia repair has become increasingly common in recent years. It is important to understand how its outcomes, particularly longer-term recurrence outcomes, compare with the other classic approaches; namely, laparoscopic and transthoracic.

Paraesophageal hernias (PEH) are estimated to represent between 5% and 30% of all hiatal hernias.^1^^,^^2^ Many patients report no symptoms, whereas others present with dysphagia, aspiration, anemia, acid reflux, early satiety, postprandial chest pain, or dyspnea. Surgery may be recommended to treat patients with refractory symptoms or asymptomatic hernias considered to be at elevated risk of volvulus, obstruction, or strangulation. Repair typically involves reduction of hernia contents, crural repair, and an antireflux intervention. There are various surgical approaches to PEH repair that have been developed and refined.^3^ Starting in the 1950s, Dr Ronald Belsey developed transthoracic repair, leading to the Belsey Mark IV fundoplication, whereas Dr Rudolph Nissen introduced the Nissen fundoplication for PEH repair and reflux treatment.^4^ In 1991, Dr Bernard Dallemagne pioneered the laparoscopic (lap) approach, later refined by Dr Alfred Cuschieri among several others.^5^^,^^6^ The lap technique has become the standard approach to PEH repair, largely due to fewer complications and a more expeditious recovery.^7^^,^^8^ Mesh has been associated with lower short-term recurrence rates but has no significant benefit for long-term rates, and carries a small risk of esophageal injury from mesh erosion.^9^ Gastropexy has also been studied as a potential intervention to decrease recurrence rates with mixed findings. Medium- and long-term radiographic recurrence after lap repair ranges from 16% to 66%,^10^, ^11^, ^12^, ^13^, ^14^, ^15^ which is higher than recurrence reported after the transthoracic repair, which is typically <10%.^8^^,^^15^, ^16^, ^17^ The lap approach is currently the most common method of PEH repair, usually performed by minimally invasive surgeons, with the transthoracic approach largely reserved for select patients with giant, complex, or reoperative cases and performed by thoracic surgeons.^8^^,^^18^ PEH repair via laparotomy is still rarely performed but has largely been replaced by the other methods, particularly for elective repairs.

Recently, robotic-assisted laparoscopic (robot) surgery for PEH repair has gained significant traction. Advantages include enhanced dexterity and visualization, which may be particularly important for high mediastinal dissection and precise crural repair, although existing evidence is limited.^8^^,^^14^^,^^19^ Concerns regarding robot technique adoption include higher cost, lack of tactile feedback, longer operative times, resource/expertise availability, and current lack of evidence for improved long-term outcomes.^8^^,^^20^, ^21^, ^22^

In this study, we directly compare outcomes between all 3 major techniques—transthoracic, lap, and robot—for PEH repair. We hypothesized that a robot approach would have lower 2-year recurrence rates compared with lap and improved short-term outcomes such as length of stay (LOS) and 30-day complications compared with transthoracic.

## Methods

### Study Design and Patient Population

A retrospective study of prospectively collected data was conducted at a single high-volume academic institution from July 2018 through September 2022. The study was approved by an institutional review board (No. HUM00047546; March 28, 2011). The institutional review board approved the study protocol and publication of data. Patient written consent for the publication of the study data was waived by the institutional review board due to the retrospective nature of the study and minimal risk to participants.

All adult patients (aged 18 years and older) who underwent primary PEH repair during the study period were included. Exclusion criteria included patients with inadequate 2-year clinical follow-up data, urgent/emergency cases, and reoperative cases. Perioperative data were collected and entered into the Society of Thoracic Surgeons (STS) database, with additional follow-up data collected via chart review. Cases were then divided into 3 groups for comparison: lap, robot, and transthoracic. All cases involved primary crural repair with 0 Ethibond (Ethicon) (lap and robot) or 1-0 silk (transthoracic) with selective biologic mesh reinforcement for the robot and lap cases. All transthoracic included a linear Collis gastroplasty with Nissen fundoplication. A Collis wedge gastroplasty was used selectively in lap cases and none in robot. Seven experienced lap and open thoracic surgeons performed all operations with all 7 performing transthoracic, 5 performing lap, and 4 performing robot procedures. All surgeons had been practicing for at least 3 years and all cases were performed with trainee involvement. The choice of surgical approach was at the discretion of the operating surgeon and reflects a historical preference for transthoracic repairs for all type 2 or greater PEH, with a gradual transition toward performing cases with lap and robot techniques. Techniques for performing lap,^23^ transthoracic,^17^ and robot^24^ procedures are described.

### Outcome Measures

Primary outcomes were all measures of recurrence by 2 years after surgery and were defined as follows: any radiographic recurrence (Rad), defined as any evidence of recurrent hiatal hernia, including small/sliding hernia on postoperative imaging or endoscopy; STS-defined radiographic recurrence (STS-rad), defined as recurrent hiatal hernia on postoperative imaging with >2 cm or 10% of stomach herniated above the diaphragm; or STS-defined symptomatic recurrence (Sx) defined as recurrence of preoperative symptoms in the context of Rad recurrence of hiatal hernia. Of note, patients who did not have 2-year imaging and had not been previously diagnosed with a recurrence were recorded as “No 2-year imaging obtained.” Secondary outcomes included total procedure time (procedure start to procedure end time or skin-to-skin as included in STS reporting), LOS, any immediate postoperative complications (included in STS reporting), postoperative endoscopic intervention (typically dilation) within 2 years, and reoperation within 2 years.

### Statistical Analysis

Analysis was performed using SAS version 9.4 (SAS Institute Inc). Median and interquartile range (IQR) were reported for nonnormally distributed variables. Means ± standard deviation were also reported for normally distributed variables. Categorical variables are reported as n (%). For comparisons among the 3 groups, continuous variables were compared using 1-way analysis of variance or Kruskal-Willis test depending on the data distribution. Categorical variables were compared using χ^2^ test or Fisher exact test with Freeman-Halton extension depending on frequencies.

We developed 3 model approaches. Each model type was adjusted for surgeon effect by treating surgeon as a cluster in the generalized estimating equations framework. We first examined univariate associations between postoperative outcomes and type of surgery. Second, we built multivariate models adjusting for age, body mass index (BMI), gender, race, American Society of Anesthesiologists (ASA) score, and hernia type. To estimate association between surgery type and binary outcomes (recurrence, endoscopic intervention, reoperation, and postoperative complication) we used a logit link and for continuous outcomes (LOS and total procedure time) we used a log link.

Third, we checked the distribution of clinical and demographic covariates across surgical approaches and found significant differences in BMI and hernia type, as well as borderline differences in ASA score and demographic factors. To account for these differences, we proceeded with propensity score analysis^25^ and built a generalized logit model to estimate the propensity to undergo each surgical approach, conditional on age, BMI, gender, race, ASA score, and hernia type. We checked the balance of covariates achieved by the model. To create a pseudopopulation balanced with respect to the covariates, we developed inverse probability of treatment weights (IPTW).^26^ Finally, to address missingness in Rad and STS-rad, we developed inverse probability weights (IPWs), defined as inverse probability of having 2-year imaging, conditional on clinical and demographic factors.^27^ We checked both IPTW and IPW and didn't find any outliers. We constructed our final weight as a product of IPTW and IPW weights. Additional information about these models can be found in Table E1 and Appendix E1.

## Results

### Patient Population

**Figure 1:**
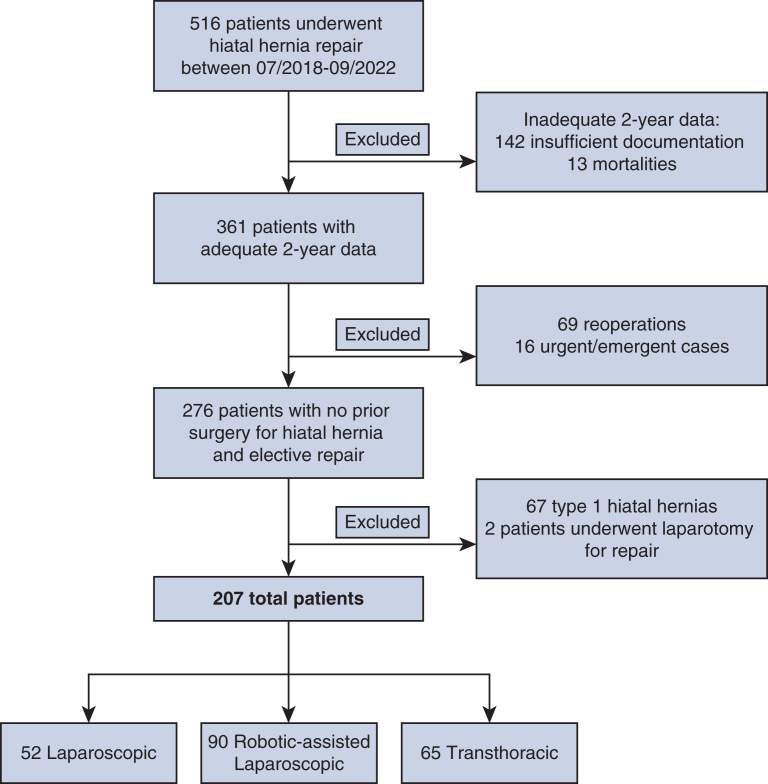
Inclusion and exclusion criteria.

A total of 516 patients underwent hiatal hernia repair surgery between July 2018 and September 2022. Patients without 2-year follow-up were excluded (155 patients, including 13 mortalities). Sixty-nine patients for whom the surgery represented a reoperation (ie, for recurrent hiatal hernia or revision of prior repair for any reason) were excluded. Sixteen patients who underwent an urgent or emergency repair were also excluded. Finally, all type I hiatal hernias were excluded (67 patients), leaving 209 patients with type II through IV PEH. Two of these patients underwent laparotomy for PEH repair; the remaining 207 were included in analyses: 52 (25%) lap, 90 (43%) robot, and 65 (31%) transthoracic repairs (Figure 1).

### Demographic and Preoperative Characteristics

Comparisons were made between groups, lap versus robot versus transthoracic, regarding demographics and preoperative characteristics (Table 1). The overall median age was 68 years and 82% of patients were women, with no significant differences between groups (*P* = .147 and *P* = .751). There were no significant differences in racial composition (*P* = .084) or insurance type between groups (*P* = .086). There were no differences in preexisting health conditions, including smoking history (*P* = .332), hypertension (*P* = .928), diabetes (*P* = .874), congestive heart failure (*P* = .730), and ASA score (*P* = .087). There was no statistically significant difference in the type of fundoplication performed, with 90% of patients overall undergoing a complete wrap (*P* = .235).

**Table 1:** Demographics and preoperative characteristics, overall and by operation performed

Demographic	All patients (N = 207)	Laparoscopic (n = 52 [25])	Robotic-assisted laparoscopic (n = 90 [43])	Transthoracic (n = 65 [31])	*P* value
Age at time of surgery (y)					
Median (Q1,Q3)	68 (61,73)	67 (59,72)	70 (62,74)	66 (61,72)	.147
Female sex	169 (82)	42 (81)	72 (80)	55 (85)	.751
BMI					**<.001**
Mean ± SD	29.6 ± 4.6	**29.4 ± 4.6**	**28.4 ± 3.6**	**31.3 ± 5.3**	
Median (IQR)	29.5 (26.0-32.2)	30.2 (26.3-32.3)	28.0 (25.7-31.3)	31.0 (28.0-35.0)	
Race					.084
White/Caucasian	194 (94)	49 (94)	88 (98)	58 (89)	
Non-White	7 (3)	0 (0)	2 (2)	5 (8)	
Not reported	5 (2)	3 (6)	0 (0)	2 (3)	
Insurance primary payor					.086
Medicare	136 (66)	29 (56)	67 (74)	40 (62)	
Commercial	45 (22)	16 (31)	16 (18)	13 (20)	
HMO	18 (9)	5 (10)	5 (6)	8 (12)	
Medicaid	7 (3)	2 (4)	2 (2)	3 (5)	
Other	1 (<1)	0 (0)	0 (0)	1 (2)	
Smoking history					.332
Current/previous∗	74 (36)	15 (29)	36 (40)	23 (35)	
Never	99 (48)	28 (54)	38 (42)	33 (51)	
Unknown	34 (16)	9 (4)	16 (18)	9 (14)	
History of hypertension	127 (61)	31 (60)	55 (61)	41 (63)	.928
History of diabetes	33 (16)	9 (17)	13 (14)	11 (17)	.874
History of congestive Heart failure	8 (4)	3 (6)	3 (3)	2 (3)	.730
ASA					.087
I	0 (0)	0 (0)	0 (0)	0 (0)	
II	73 (35)	22 (42)	35 (39)	16 (25)	
III	132 (64)	30 (58)	54 (60)	48 (74)	
IV	2 (1)	0 (0)	1 (1)	1 (2)	
Hiatal hernia type					**.009**
II	5 (2)	**1 (2)**	**2 (2)**	**2 (3)**	
III	178 (86)	**46 (88)**	**83 (92)**	**49 (75)**	
IV	26 (13)	**5 (10)**	**5 (6)**	**14 (22)**	
Type of fundoplication					.235
Complete	187 (90)	45 (87)	80 (89)	62 (95)	
Partial	16 (8)	5 (10)	9 (10)	2 (3)	
None†	4 (2)	2 (4)	1 (1)	1 (2)	
Mesh use	21 (10)	**19** (**37)**	**2** (**2)**	**0** (**0)**	**<.001**

Values are presented as n (%) unless otherwise noted. Statistically significant findings are presented in bold.

*BMI*, Body mass index; *SD*, standard deviation; *IQR*, interquartile range; *HMO*, health maintenance organization; *ASA*, American Society of Anesthesiologists.

∗ Three current smokers, all others previous.

† Gastropexy performed or history of Roux-en-Y gastric bypass or esophagectomy.

There was a statistically significant difference between groups in average BMI, with transthoracic having the highest BMI and robot the lowest (lap: 29.4, robot: 28.4, transthoracic: 31.3; *P* < .001). Additionally, there was a significant difference in the type of hiatal hernia being repaired. Type II hiatal hernia were rare with only 5 total. Transthoracic had 75% type 3 hiatal hernias and 22% type IV hiatal hernias, lap had 88% type III hiatal hernias and 10% type 4 hiatal hernias, and robot had 92% type 3 hiatal hernias and 6% type IV hiatal hernias (*P* = .009). Finally, there was a much higher rate of mesh use in lap (37%) compared with robot (2%) and transthoracic (0%; *P* < .001).

### Perioperative and Secondary Outcomes by Operation

#### Total procedure time

Overall median procedure time was 186 minutes (IQR, 150-222 minutes). Lap median procedure time was 186 minutes (IQR, 140-236 minutes), robot median procedure time was 178 minutes (IQR, 150-210.5 minutes), and transthoracic median procedure time was 200 minutes (IQR, 170-241 minutes). Across analyses, there were no significant differences between surgical approaches (Table 2).

**Table 2:** Secondary outcomes by operation performed

Demographic	Univariate analysis	Multivariate analysis	Propensity score analysis
Total procedure time	Change in least squares mean (95% CI)		
Laparoscopic vs transthoracic	−0.12 (−0.42 to 0.17)	−0.11 (−0.45 to 0.23)	−0.12 (−0.45 to 0.21)
Robotic vs laparoscopic	0.09 (−0.15 to 0.33)	0.08 (−0.15 to 0.32)	0.02 (−0.19 to 0.23)
Robotic vs transthoracic	−0.03 (−0.21 to 0.15)	−0.02 (−0.27 to 0.22)	−0.10 (−0.34 to 0.14)
Length of stay	% Change in least squares mean (95% CI)		
Laparoscopic vs transthoracic	**−0.94** (**–1.29 to –0.60)**†	**−0.89 (–1.30 to –0.49)**†	**−0.86 (–1.20 to –0.52)**†
Robotic vs laparoscopic	−0.15 (−0.52 to 0.23)	−0.09 (−0.52 to 0.34)	−0.16 (−0.52 to 0.19)
Robotic vs transthoracic	**−1.09 (–1.23 to –0.95)**†	**−0.98 (–1.15 to –0.81)**†	**−1.02 (–1.21 to –0.84)**†
Any immediate postoperative complication	Odds ratio (95% CI)		
Laparoscopic vs transthoracic	**0.30 (0.21 to 0.41)**†	**0.27 (0.21 to 0.34)**†	**0.41 (0.29 to 0.59)**†
Robotic vs laparoscopic	0.64 (0.40 to 1.02)	0.71 (0.49 to 1.04)	**0.51 (0.33 to 0.80)**†
Robotic vs transthoracic	**0.19 (0.10 to 0.35)**†	**0.19 (0.11 to 0.34)**†	**0.21 (0.12 to 0.39)**†
Endoscopic intervention (2-y)			
Laparoscopic vs transthoracic	1.73 (0.70 to 4.30)	2.21 (0.83 to 5.88)	2.16 (0.88 to 5.29)
Robotic vs laparoscopic	**0.40 (0.21 to 0.76)**†	**0.37 (0.17 to 0.85)**∗	**0.32 (0.16 to 0.64)**†
Robotic vs transthoracic	0.69 (0.25 to 1.92)	0.83 (0.28 to 2.45)	0.70 (0.23 to 2.15)
Reoperation (2-y)			
Laparoscopic vs transthoracic	12.18 (0.70 to 210.6)	11.00 (0.47 to 258.8)	5.56 (0.64 to 48.66)
Robotic vs laparoscopic	**0.12 (0.05 to 0.30)**†	**0.09 (0.03 to 0.31)**†	**0.10 (0.04 to 0.25)**†
Robotic vs transthoracic	1.47 (0.04 to 61.45)	1.01 (0.01 to 77.38)	0.53 (0.02 to 11.53)

Statistically significant findings are presented in bold.

∗ *P* < .05.

† *P* < .01.

#### LOS

The overall median LOS was 3 days (IQR, 1-5 days). Lap LOS was 2 days (IQR, 1-3 days), robot LOS was 2 days (IQR, 1-2 days), and transthoracic LOS was 5 days (IQR, 4-6 days). Across all three methods of analysis (Table 2), lap had a shorted LOS compared with transthoracic (*P* < .01) and robot had a shorter LOS compared with transthoracic (*P* < .01), but there were no significant differences between robot and lap.

#### Immediate postoperative complication

The overall rate of any immediate postoperative complication was 27% (56 out of 207). Lap postoperative complication rate was 19% (10 out of 52), robot was 17% (15 out of 90), and transthoracic was 48% (31 out of 65). In univariate, multivariate, and propensity score analyses, lap and robot postoperative complication rates were both significantly lower than transthoracic (odds ratio [OR], 0.27-0.41; *P* < .01 and OR, 0.19-0.21; *P* < .01) (Table 2), In propensity score analysis only, robot had a lower postoperative complication rate compared with lap (OR, 0.51; *P* < .01), but univariate and multivariate comparisons were not significant.

#### Endoscopic intervention

The overall rate of endoscopic intervention (dilation) by years was 13% (26 out of 207). Lap had 19% (10 out of 52), robot had 9% (8 out of 90), and transthoracic had 12% (8 out of 65). Robot had significantly lower endoscopic intervention compared with lap across all 3 methods of comparison (OR, 0.32-0.40; *P* < .05) (Table 2). No other significant differences were found between groups (Table 2).

#### Reoperation

The overall rate of reoperation within 2 years was 4% (9 out of 207). Lap reoperation rate was 10% (5 out of 52), robot 3% (3 out of 90), and transthoracic 2% (1 out of 65). Robot had significantly lower rates of reoperation compared with lap in univariate analysis across all 3 analyses (OR, 0.09-0.12; *P* < .01) (Table 2). Otherwise, no significant differences were found between groups across analyses (Table 2).

### Primary 2-Year Recurrence Outcomes by Operation

#### Rad recurrence

The overall 2-year Rad rate among all patients regardless of 2-year imaging completion status was 44% (91 out of 207). Lap Rad was 73% (38 out of 52), robot Rad was 37% (33 out of 90), and transthoracic Rad was 31% (20 out of 65). Across univariate and multivariate analyses, lap had a higher Rad rate compared with transthoracic (OR, 5.95-5.95; *P* < .01), robot had a lower Rad rate compared with lap (OR, 0.13-0.17; *P* < .01), and robot had no significant difference compared with transthoracic (OR, 0.79-1.02; *P* > .05) (Table 3). There were no differences between approaches on propensity score analysis.

**Table 3:** Primary 2-year recurrence outcomes by operation performed

Demographic	Univariate analysis, OR (95% CI)	Multivariate analysis, OR (95% CI)	Propensity score analysis, OR (95% CI)
Rad recurrence (n = 152)			
Laparoscopic vs transthoracic	**5.95** (**1.80-19.70)**∗	**5.95 (2.04-17.36)**∗	5.70 (0.83-39.36)
Robotic vs laparoscopic	**0.17** (**0.05-0.59)**∗	**0.13 (0.03-0.52)**∗	0.15 (0.02-1.24)
Robotic vs transthoracic	1.02 (0.66-1.59)	0.79 (0.59-1.06)	0.84 (0.46-1.57)
STS-rad recurrence (n = 139)			
Laparoscopic vs transthoracic	2.47 (0.83-7.34)	2.92 (0.95-5.26)	1.44 (0.69-2.99)
Robotic vs laparoscopic	0.68 (0.28-1.62)	0.45 (0.19-1.05)	1.27 (0.66-2.46)
Robotic vs transthoracic	1.67 (0.87-3.21)	1.31 (0.53-3.21)	**1.83 (1.50-2.24)**∗
Sx recurrence (n = 207)			
Laparoscopic vs transthoracic	**3.91 (1.52-10.08)**∗	**4.74 (2.05-10.98)**∗	**4.08 (2.17-7.64)**∗
Robotic vs laparoscopic	**0.40 (0.26-0.63)**∗	**0.50 (0.34-0.74)**∗	**0.43 (0.35-0.53)**∗
Robotic vs transthoracic	1.57 (0.66-3.75)	**2.37 (1.35-4.19)**∗	1.76 (0.91-3.42)

Statistically significant findings are presented in bold.

*OR*, O*dds ratio; Rad*, radiographic; *STS-rad*, STS-defined radiographic; *Sx*, symptomatic.

∗ *P* < .01.

#### STS-rad recurrence

The overall 2-year STS-rad recurrence among all patients regardless of 2-year imaging completion status was 19% (39 out of 207). Lap STS-rad was 31% (16 out of 52), robot STS-rad was 18% (16 out of 90), and transthoracic STS-rad was 11% (7 out of 65). There were no significant differences between approached across univariate, multivariate, and propensity score analyses with the only exception being robot had a significantly higher STS-rad compared with transthoracic in propensity score analysis (OR, 1.83; *P* < .01) (Table 3).

#### Sx recurrence

**Figure 2:**
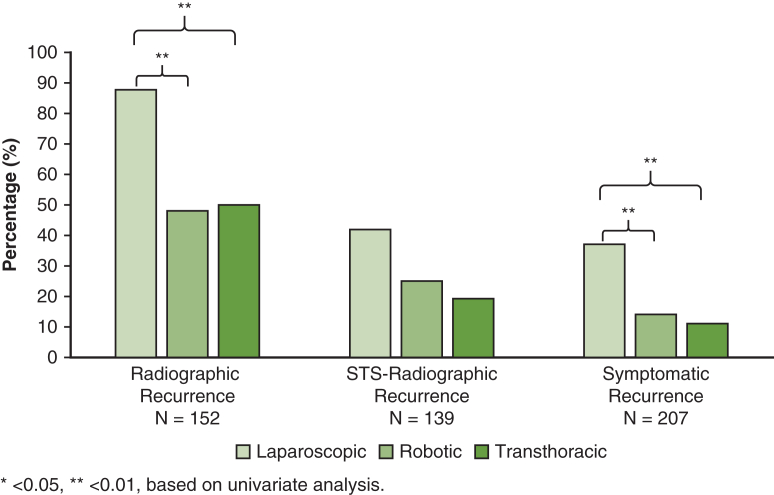
Two-year recurrence by operation. Based on univariate analysis, with laparoscopic being compared with transthoracic; robotic-assisted laparoscopic being compared with laparoscopic, and transthoracic being compared with robotic-assisted laparoscopic. *STS*, Society of Thoracic Surgeons.

The overall 2-year Sx rate was 19% (29 out of 207). Lap Sx was 37% (19 out of 52), robot was 14% (13 out of 90), and transthoracic was 11% (7 out of 65) (Figure 2). Across all 3 methods of comparison, lap had a higher Sx rate compared with transthoracic (OR, 3.91-4.74; *P* < .01), and robot had a lower Sx rate compared with lap (OR, 0.40-0.50; *P* < .01) (Table 3). Robot had a higher rate of Sx compared with transthoracic in multivariate analysis (OR, 2.37; *P* < .01), but not in univariate or propensity score analyses (Table 3).

## Discussion

This study compares perioperative and long-term outcomes of the 3 most common approaches for PEH repair with a focus on 2-year recurrence. Recurrence rates for robot repairs were lower compared with lap and similar to transthoracic across most recurrence definitions and analyses. There are few studies comparing long-term recurrence between robot and other operative approaches.^8^^,^^22^^,^^28^ A 2023 systematic review by Elissavet and colleagues^22^ compared robot and lap perioperative outcomes but was unable to assess recurrence due to lack of available data from published studies. Robot recurrence rates have been reported between 2.5% and 42%, with follow-ups between 1 month and 5 years and varying definitions of recurrence.^19^^,^^22^ O'Connor and colleagues^14^ found lower 1-year recurrence rates after robot repair compared with lap (13.3% vs 32.8%) despite more redo PEH repairs in the robotic group. However, there was longer follow-up in the lap group. We specifically excluded reoperations from our study to eliminate this as a potential confounder. There are limited data directly comparing robot and transthoracic recurrence rates, although transthoracic repair has historically shown recurrence rates under 10%.^8^^,^^15^, ^16^, ^17^ Our institutional approach had historically been to perform transthoracic repairs without using mesh. As different faculty with different training joined our group, we diversified our approaches, with the use of biologic mesh occasionally, mostly with the lap repairs. We currently still perform all modalities of repair but favor the transthoracic and robot/lap approaches based on our experiences. We speculate that the robot approach allows mobilization of the mediastinal esophagus to the subcarinal, providing more intra-abdominal esophageal length later to reduce the risk of recurrence.

Our study also highlights how varying definitions for PEH recurrence can have a large impact on reporting. We report lap 2-year recurrence between 31% and 73%, depending on the recurrence definition used. Lap recurrence rates reported in the literature at similar follow-up durations range from 20% to 40%,^12^^,^^14^^,^^29^ with longer follow-up durations (ie, 5 years+) often up to >60%.^9^, ^10^, ^11^ Many recurrent hiatal hernias identified under our broadest recurrence definition were small, sliding, asymptomatic hernias with intact wraps, arguably of low clinical significance. In general, the significance of radiographic recurrence has been questioned. A definition of >2 cm of stomach herniated on follow-up imaging has been proposed and used by some as a standard definition for Rad because <2 cm recurrences rarely required reoperation and report good quality of life outcomes.^29^^,^^30^ Alternatively, Braghetto and colleagues^31^ proposed a scoring system to define clinically significant recurrence based on symptoms and imaging. Although symptomatic recurrence is more patient-centered, its measurement is challenging due to symptom variability and potential alternative causes like stenosis. Our definition of symptomatic recurrence necessitated radiographic evidence of recurrence to control for some of these factors.

Robot surgery had the shortest median procedure time of 178 minutes; 22 minutes shorter than transthoracic and 8 minutes shorter than lap, although these differences were not significant. Although robot procedures were once considered longer,^32^ recent studies report similar^14^^,^^21^^,^^22^ or even shorter^19^ times compared with laparoscopic; perhaps a result of increased surgeon experience.^8^ We argue that the small differences in procedure times seen in our study lack clinical significance. Median LOS after robot or lap repair was 2 days, significantly shorter compared with 5 days in the transthoracic group, which is consistent with prior studies.^14^^,^^19^^,^^22^^,^^28^^,^^32^

Thirty-day postoperative complications after robotic PEH repair were significantly lower than transthoracic, and similar or lower compared with laparoscopic. This may be secondary to increased respiratory and cardiac manipulation and increased recovery associated with thoracotomy compared with a minimally invasive transabdominal approach. Meta analyses by Elissavet and colleagues^22^ and Bhatt and Wei^28^ found no significant difference in perioperative complications between robot and lap repairs. However, 1 large study in the early years of robotics demonstrated a higher rate of esophageal perforation and respiratory failure in the robotic group,^28^ which may again point toward a robotics learning curve.

Robot repair had lower rates compared with lap of endoscopic intervention (9% vs 19%) and reoperation (3% vs 10%) at 2 years postoperation. A meta-analysis by Tasoudis and colleagues^16^ and others^15^ found significantly higher rates of reoperation following lap Nissen repair compared with transthoracic Belsey. Transthoracic approach has low reoperation rates (<5%) even with large hernias, which is consistent with our findings.^15^, ^16^, ^17^ Long-term reoperation comparison between lap and robot approaches has not previously been studied.

Limitations of this study include demographic and hiatal hernia type differences between groups, with patients in the robotic group having a lower BMI and the transthoracic group having more type IV hiatal hernias, likely a result of surgeon preference. Also, surgeon preference, varying levels of experience, and improved surgical experience over time all may contribute to selection bias. Approximately 30% of patients were missing 2-year imaging, and this was highest in the transthoracic group, which could result in differential underdetection in radiographic recurrence rates. Statistical expertise consultation and censoring weights to account for patients missing 2-year imaging were executed in an effort to account for this. Finally, several outcomes and other important measures in determining surgical approach are outside of the scope of this study and should be a focus in future work. This includes costs considerations and patient experience measures.

## Conclusions

At our institution, robot PEH generally had lower 2-year Rad, Sx, endoscopic intervention, and reoperation compared with lap. Robot repairs had shorter hospital stays and fewer immediate complications compared with transthoracic. Selection bias is a major limitation of this retrospective study. The robot approach may offer optimal benefits of low short-term complications rates and lower long-term recurrence rates.

### Declaration of Generative AI and AI-Assisted Technologies in the Writing Process

During the preparation of this work the authors used ChatGPT4o for grammar review and suggestions for language improvement and clarity After using this tool/service, the author(s) reviewed and edited the content as needed and take(s) full responsibility for the content of the publication.

### Webcast

You can watch a Webcast of this AATS meeting presentation by going to: https://www.aats.org/resources/robotic-laparoscopic-paraesoph-9484.

**Figure:**
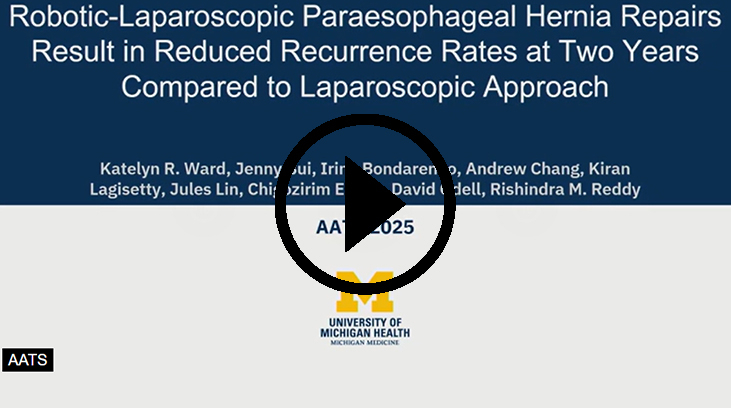

## Conflict of Interest Statement

Dr Lagisetty has received a research grant from Atricure. Dr Ekeke is a consultant with Atricure and Cook Medical and on the advisory board of Ethicon/JNJ. Dr Odell has received grants from the Agency for Healthcare Research and Quality. Dr Reddy is a teaching site/consultant with Intuitive Surgical, has received grants from and is on the advisory board of Atricure and On Target Laboratories, is on the advisory board of Medtronic and Genentech, is a speaker with BMS, and is a consultant with Trinity Health. All other authors reported no conflicts of interest.

The *Journal* policy requires editors and reviewers to disclose conflicts of interest and to decline handling or reviewing manuscripts for which they may have a conflict of interest. The editors and reviewers of this article have no conflicts of interest.

## Figures and Tables

**Table E1:** Multivariable model predictors∗

Demographic	Radiological recurrence		STS recurrence	
	Odds ratio (95% CI)	*P* value	Odds ratio (95% CI)	*P* value
Laparoscopic vs thoracic	5.45 (1.76-16.88)	.0032	2.92 (0.98-8.69)	.0535
Laparoscopic vs thoracic	0.73 (0.29-1.81)	.4980	1.31 (0.45-3.80)	.6248
BMI	0.94 (0.85-1.03)	.1825	0.98 (0.89-1.08)	.7087
HH_type = III vs II	0.37 (0.01-22.84)	.6345	2.90 (0.06-141.7)	05,912
HH_type = IV vs II	0.38 (0.01-26.58)	.6526	3.67 (0.07-201.7)	.5251
Age	0.99 (0.96-1.03)	.6706	1.01 (0.97-1.04)	.7875
ASA III/IV vs ASA = II	0.80 (0.36-1.81)	.5985	0.73 (0.31-1.72)	.4762
Female vs male	1.40 (0.54-3.61)	.4998	1.17 (0.42-3.25)	.7657
Race (White vs non-White)	1.75 (0.38-8.10)	.4735	2.02 (0.27-15.26)	.4960

*STS*, Society of Thoracic Surgeons; *BMI*, body mass index; *HH*, hiatal hernia; *ASA*, American Society of Anesthesiologists.

∗ Approach was included in the multivariable model as a multi-level variable with transthoracic as the control.

## Appendix E1. Propensity Model Additional Information

We first used a generalized logit model to estimate propensity scores for the probability of treatment: p_k = (P(Surgery type = )k| age, body mass index, American Society of Anesthesiologists score, type of hernia, race, and gender). Each subject was assigned a probability of actual treatment. We used this propensity to construct the inverse probability of treatment weights (IPTW), defined as the reciprocal of the propensity of treatment:

w_iptw = 1/p_k.

Additionally, for outcomes with missingness in the outcome (Society of Thoracic Surgeons and radiological recurrence) we developed a weight for missingness w_m_j = 1/P(outcome_j = observed| type of surgery, age, body mass index, American Society of Anesthesiologists score, type of hernia, race, and gender). For the model with completely observed outcomes, w_m was defined as w_m_j = 1.

The final weight for outcome j was defined as F_w_j = w_iptw∗w_m_j.

## References

[1] Kohn G.P., Price R.R., DeMeester S.R. (2013). Guidelines for the management of hiatal hernia. Surg Endoscop.

[2] Kim J., Hiura G.T., Oelsner E.C. (2021). Hiatal hernia prevalence and natural history on non-contrast CT in the Multi-Ethnic Study of Atherosclerosis (MESA). BMJ Open Gastroenterol.

[3] Stylopoulos N., Rattner D.W. (2005). The history of hiatal hernia surgery: from Bowditch to laparoscopy. Ann Surg.

[4] Trus T.L., Grams J., Perry K.A., Tavakkoli A. (2019). The SAGES Manual of Foregut Surgery.

[5] Cuschieri A., Shimi S., Nathanson L.K. (1992). Laparoscopic reduction, crural repair, and fundoplication of large hiatal hernia. Am J Surg.

[6] Rajkomar K., Berney C.R. (2022). Large hiatus hernia: time for a paradigm shift?. BMC Surg.

[7] McLaren P.J., Hart K.D., Hunter J.G., Dolan J.P. (2017). Paraesophageal hernia repair outcomes using minimally invasive approaches. JAMA Surg.

[8] Fallon B.P., Reddy R.M. (2021). Choosing the best approach for paraesophageal hiatal hernia repair: a narrative review. Video Assist Thorac Surg.

[9] Oelschlager B.K., Pellegrini C.A., Hunter J.G. (2011). Biologic prosthesis to prevent recurrence after laparoscopic paraesophageal hernia repair: long-term follow-up from a multicenter, prospective, randomized trial [published correction appears in *J Am Coll Surg.* 2011;213(6):815]. J Am Coll Surg.

[10] Morrow E.H., Oelschlager B.K. (2013). Laparoscopic paraesophageal hernia repair. Surg Laparosc Endosc Percutan Tech.

[11] Dallemagne B., Kohnen L., Perretta S. (2011). Laparoscopic repair of paraesophageal hernia. Long-term follow-up reveals good clinical outcome despite high radiological recurrence rate. Ann Surg.

[12] Nguyen C.L., Tovmassian D., Zhou M. (2023). Recurrence in paraesophageal hernia: patient factors and composite surgical repair in 862 cases. J Gastrointest Surg.

[13] Antiporda M., Veenstra B., Jackson C. (2018). Laparoscopic repair of giant paraesophageal hernia: are there factors associated with anatomic recurrence?. Surg Endosc.

[14] O'Connor S.C., Mallard M., Desai S.S. (2020). Robotic versus laparoscopic approach to hiatal hernia repair: results after 7 years of robotic experience. Am Surg.

[15] Laan D.V., Agzarian J., Harmsen W.S. (2018). A comparison between Belsey Mark IV and laparoscopic Nissen fundoplication in patients with large paraesophageal hernia. J Thorac Cardiovasc Surg.

[16] Tasoudis P., Vitkos E., Haithcock B.E., Long J.M. (2023). Transthoracic fundoplication using the Belsey Mark IV technique versus Nissen fundoplication: a systematic review and meta-analysis. Surg Endosc.

[17] Patel H.J., Tan B.B., Yee J., Orringer M.B., Iannettoni M.D. (2004). A 25-year experience with open primary transthoracic repair of paraesophageal hiatal hernia. J Thorac Cardiovasc Surg.

[18] Migliore M., Arcerito M., Vagliasindi A. (2003). The place of Belsey Mark IV fundoplication in the era of laparoscopic surgery. Eur J Cardiothorac Surg.

[19] Gerull W.D., Cho D., Kuo I. (2020). Robotic approach to paraesophageal hernia repair results in low long-term recurrence rate and beneficial patient-centered outcomes. J Am Coll Surg.

[20] Tonelli C.M., Baker M.S., Luchette F.A., Cohn T. (2023). Laparoscopic and robotic paraesophageal hernia repair in United States veterans: clinical outcomes and risk factors associated with reoperation recurrence. Am J Surg.

[21] Lekarczyk A., Sinha H., Dvir D. (2023). Similar hospital profits with robotic-assisted paraesophageal hiatal hernia repair, despite higher or supply costs. Surg Endosc.

[22] Elissavet S., Ioannis G., Panagiotis P., Konstantinos M., Apostolos K. (2023). Robotic-assisted versus laparoscopic paraesophageal hernia repair: a systematic review and meta-analysis. J Minim Invasive Surg.

[23] Carrott P.W. (2014). Laparoscopic paraesophageal hiatus hernia repair. Operat Techniq Thorac Cardiovasc Surg.

[24] Ekeke C.N., Vercauteren M., Baker N., Sarkaria I. (2019). Surgical techniques for robotically-assisted laparoscopic paraesophageal hernia repair. Thorac Surg Clin.

[25] Rosenbaum P.R., Rubin D.B. (1983). The central role of the propensity score in observational studies for causal effects. Biometrika.

[26] Austin P.C., Stuart E.A. (2015). Moving towards best practice when using inverse probability of treatment weighting (IPTW) using the propensity score to estimate causal treatment effects in observational studies. Stat Med.

[27] Seaman S.R., White I.R. (2013). Review of inverse probability weighting for dealing with missing data. Stat Methods Med Res.

[28] Bhatt H., Wei B. (2023). Comparison of laparoscopic vs. robotic paraesophageal hernia repair: a systematic review. J Thorac Dis.

[29] Lidor A.O., Kawaji Q., Stem M. (2013). Defining recurrence after paraesophageal hernia repair: correlating symptoms and radiographic findings. Surgery.

[30] Oelschlager B.K., Petersen R.P., Brunt L.M. (2012). Laparoscopic paraesophageal hernia repair: defining long-term clinical and anatomic outcomes. J Gastrointest Surg.

[31] Braghetto I., Lanzarini E., Musleh M., Korn O., Lasnibat J.P. (2018). Thinking about hiatal hernia recurrence after laparoscopic repair: when should it be considered a true recurrence? A different point of view. Int Surg.

[32] Soliman B.G., Nguyen D.T., Chan E.Y. (2020). Robot-assisted hiatal hernia repair demonstrates favorable short-term outcomes compared to laparoscopic hiatal hernia repair. Surg Endosc.

